# Ein mikrochirurgisches Wetlab für Studierende steigert das Interesse an der Augenheilkunde

**DOI:** 10.1007/s00347-020-01079-5

**Published:** 2020-03-11

**Authors:** Andreas Müller, Norbert Pfeiffer, Franziska Schmidt, Verena Prokosch

**Affiliations:** 1 Augenklinik und Poliklinik der Universitätsmedizin, Johannes Gutenberg-Universität Mainz, Langenbeckstr. 1, 55131 Mainz, Deutschland; 2 Zentrums für Qualitätssicherung und -entwicklung, Johannes Gutenberg Universität Mainz, Mainz, Deutschland

**Keywords:** Studium, Nachwuchsmangel, Praktischer Kurs, Weiterbildung, Facharztwahl, Medical school, Eye medicine, Practical course, Specialisation, Trainees

## Abstract

**Hintergrund:**

Praktischen Inhalten wird in den Curricula der Humanmedizin zunehmend Platz eingeräumt. Bei verbreitetem Bewerbermangel trägt dies zu einem vermehrten Interesse seitens der Studierenden an den jeweiligen Fachbereichen bei. Einen praktischen Reiz der Augenheilkunde stellt die mikrochirurgische Arbeitsweise dar. Eine Einführung kann beispielsweise mit einem mikrochirurgischen Nahtkurs geleistet werden.

**Ziel der Arbeit:**

Erfassung des Zugewinns des Interesses an der Augenheilkunde mittels Evaluation eines Nahtkurs-Wetlabs inklusive Nähen unter dem Mikroskop.

**Material und Methoden:**

Die Daten wurden im Blockpraktikum Augenheilkunde des 6. Semesters an der Universitätsmedizin Mainz im April 2019 erhoben. In einem Fragebogen wurden verschiedene Aussagen auf Ordinalskalen gemeinsam mit dem Zentrum für Qualitätssicherung und -entwicklung der Johannes-Gutenberg-Universität Mainz bewertet und ausgewertet.

**Ergebnisse:**

Es wurden 64 Evaluationsbögen von 8 Gruppen unterschiedlicher Dozenten zu je 8 Teilnehmern ausgewertet. Das Wetlab wurde im Mittel mit einer Schulnote von 1,24 ± 0,5 (MW±SD) bewertet. Es bestand Zustimmung (1 = stimme völlig zu, 7 = stimme gar nicht zu) zum Wunsch nach weiteren Wetlabs unter dem Mikroskop (1,86 ± 1,28) sowie nach dem Erlernen von mehr augenchirurgischen Techniken (2,02 ± 1,13). Das Interesse an der Augenheilkunde (1 = sehr groß, 7 = sehr gering) nahm von 3,66 ± 1,55 zu Beginn des Kurses auf 2,52 ± 1,00 zu.

**Diskussion:**

Das Interesse an der Augenheilkunde lässt sich mittels eines mikrochirurgischen Wetlabs steigern. Bei Studierenden kann so das Interesse an der Augenheilkunde geweckt werden, was sich vorteilhaft auf Bewerbersituation und Forschungsarbeiten auswirken kann. So können sich bereits im Studium Erfahrungen und praktische Techniken der Augenheilkunde angeeignet werden.

## Abstract (en)

**Background:**

Practical contents are gaining in importance in medical curricula in Germany. In times of a lack of applicants, practical courses provide an increased level of interest for students in the respective disciplines. A practical appeal of ophthalmology is microsurgical procedures. A microsurgical suture wet lab can be an introduction for medical students.

**Objective:**

Assessment of the increased interest in ophthalmology through evaluation of a suture wet lab course, including suturing under a microscope.

**Material and methods:**

The data were obtained from the practical course in ophthalmology during the sixth semester for medical students at the university medical center in Mainz in April 2019. In a questionnaire developed in collaboration with the Center for Quality Assurance and Development various statements had to be answered and evaluated using ordinal scales.

**Results:**

A total of 64 evaluation questionnaires from 8 groups each with 8 medical students supervised by different tutors were analyzed. The wet lab was rated with 1.24 ± 0.5 (mean ± SD) on a German school grade scale (1 = best, 6 = worst). There was agreement (1 = total agreement, 7 = total disagreement) concerning the desire for further microscopic wet labs (1.86 ± 1.28) as well as learning more ophthalmological surgical techniques (2.02 ± 1.13). The interest in ophthalmology (1 = very high interest, 7 = very low interest) increased from 3.66 ± 1.55 before the course to 2.52 ± 1.00.

**Conclusion:**

Interest in ophthalmology can be increased through a microsurgical wet lab. The interest of students can therefore be awakened, which can have advantageous effects in job application situations and in research work. In this way experience and practical skills in ophthalmology can already be acquired during medical studies.

Eine attraktive Lehre kann eine bedeutende Rolle in der Berufsorientierung von Medizinstudierenden spielen. Um in der Konkurrenz um Bewerber nicht den Anschluss zu verlieren, muss sich auch in der Augenheilkunde um moderne Lehrkonzepte bemüht werden, besonders in Zeiten von Bewerbermangel [4]. Hierbei besteht die Möglichkeit, einen der Anreize des Faches hervorzuheben: die Anforderungen an manuelles Geschick und Präzision. Dies taten wir in einem Wetlab für Studierende und führten eine Evaluation durch, um einen möglichen Interessenszuwachs an einer zukünftigen Tätigkeit als Augenarzt zu überprüfen.

Der Facettenreichtum der Augenheilkunde fordert den Ophthalmologen in zahlreichen Kompetenzen heraus und macht das Fach abwechslungsreich und vielseitig. Auch die medizinische Lehre soll im Rahmen einer zeitgemäßen Ausbildung vielseitiger werden: Kompetenzerwerb, nicht nur in Wissenschaftlichkeit oder Kommunikation, sondern gerade auch in praktischen Fähigkeiten soll neben dem klinisch-theoretischen Fachwissen an Stellenwert deutlich zunehmen [8].

Derzeit besteht in der Augenheilkunde wohl einer der ausgeprägtesten Bewerbermangel aller Fachdisziplinen [4]. Um in dem Wettbewerb um vielversprechende Studierende nicht ins Hintertreffen zu geraten, ist eine moderne und praxisnahe Ausbildung notwendig. Die allgemeinmedizinische Lehre geht mit einer solchen Ausbildung in ihrer Nachwuchssuche erfolgreich voran [2, 10].

In den ophthalmologischen Modulen vieler universitärer Häuser werden die mitunter spannendsten Aspekte der Augenheilkunde nur passiv vermittelt. So mag zwar die Hospitation im Operationssaal für die Studierenden interessante Eindrücke in Bezug auf die mikrochirurgischen Erfordernisse des menschlichen Auges ermöglichen, die tatsächliche Durchführung von Operationsschritten unter einem Mikroskop bleibt jedoch für den Zuschauer abstrakt.

Um aus der abstrakten Faszination für Mikrochirurgie eine konkrete Begeisterung für die klinisch-praktischen Aspekte der Augenheilkunde zu wecken, führten wir im Rahmen unseres Blockpraktikums ein Wetlab mit einem mikrochirurgischen Nahtkurs durch. Hierbei erhofften wir uns zum einen, den Studierenden Nahttechniken als eine allgemein wichtige Grundlage beizubringen, zum anderen aber den besonderen Kontext, in dem diese in der Augenheilkunde stattfinden, zu betonen.

Unser Ziel war, das Interesse der Studierenden am Fachgebiet der Augenheilkunde mittels des Blockpraktikums zu steigern und das angebotene Wetlab durch die Studierenden evaluieren zu lassen.

## Methodik

Es nahmen 64 Teilnehmer im 6. Fachsemester am verpflichtenden Blockpraktikum Augenheilkunde der Poliklinik und Augenklinik der Universitätsmedizin Mainz im April 2019 teil. Die Teilnehmer wurden aufgefordert, nach Abschluss des Blockpraktikums einen Evaluationsbogen auszufüllen, welcher in Zusammenarbeit mit dem Zentrum für Qualitätssicherung und -entwicklung (ZQ) der Johannes-Gutenberg-Universität Mainz gestaltet wurde. Auf diesem wurden zum Wetlab sowie zur gesamten Blockveranstaltung Schulnoten vergeben und auf Likert-Skalen Aussagen zur Veranstaltung und der Einschätzung des individuellen Interesses an der Augenheilkunde bewertet. Weiterhin konnten Freitextantworten verfasst werden. Das ZQ wertete die Fragebögen anschließend aus. Es wurde weiterhin ein zweiseitiger Wilcoxon-Test durchgeführt, um den Interessenszuwachs durch das Blockpraktikum statistisch zu prüfen. Hierfür wurde MedCalc (version 16.8.4.0, Medcalc Software Ltd, Ostend, Belgien) verwendet.

### Aufbau des Blockpraktikums und Wetlabs

Der praktische Kursabschnitt der Augenheilkunde im 6. Semester des Humanmedizinstudiums an der Universitätsmedizin Mainz wird entweder während der Vorlesungszeit (ohne Wetlab über mehrere Kurstage) oder in der vorlesungsfreien Zeit über 2 Tage als neues „Blockpraktikum“ mit Wetlab angeboten. Letzteres ist Gegenstand der Evaluation gewesen.

Eingeführt wurde das neue Blockpraktikum, da aufgrund des Zuwachses der Studierenden je Semester eine Durchführung des herkömmlichen Praktikums während der Vorlesungszeit kapazitätsmäßig nicht mehr möglich war. Die Studierenden können sich für das Blockpraktikum (statt des herkömmlichen Praktikums) freiwillig melden, vakant verbleibende Teilnehmerplätze werden jedoch auch ohne freiwillige Meldung zugeteilt, um eine Durchführung des Kurses für alle Studierenden des Semesters zu gewährleisten.

An den Blockpraktikumstagen gibt es eine Vor- und eine Nachmittagsgruppe. Die Gesamtdauer des Unterrichts beträgt 9 h (2-mal 4 1/2 h). Die Gruppengröße beträgt 8 Teilnehmer je Gruppe mit einem augenärztlichen Betreuer.

Der erste Tag des Blockpraktikums besteht aus kurzen Vorträgen mit beispielhaften Befunden zu Pathologien des vorderen und hinteren Augenabschnittes, einer Wiederholung der Untersuchungstechnik an der Spaltlampe (primär wird diese bereits im vorherigen 5. Semester erlernt) sowie einer eigenständigen Untersuchung von insgesamt 4 Patienten der Augenklinik, 2 zum Thema vorderer, 2 zum Thema hinterer Augenabschnitt. Für die Patientenuntersuchung wird die Gruppe in 2 Gruppen von jeweils 4 Studierenden unterteilt. Die Patientenfälle werden abschließend in der vollständigen Gruppe mit Betreuer besprochen.

Am zweiten Tag finden eine Hospitation im Augen-OP sowie das mikrochirurgische Naht-Wetlab statt, welche jeweils gute 2 h umfassen. Das Wetlab unterteilt sich in 2 Abschnitte: Im ersten werden grundlegende chirurgische Nahttechniken (z. B. Einzelknopfnaht) vorgestellt und praktisch eingeübt. Im zweiten Abschnitt werden die erlernten Knüpftechniken in mikrochirurgischem Setting (entsprechendes Nahtmaterial und Instrumente) an einem mobilen Mikroskop erprobt. Je nach individuellem Kenntnisstand und Geschick können Haut‑, Lid- und Hornhautnähte mikroskopisch unter Anleitung des Betreuers durchgeführt werden. Auch hierbei steht ein betreuender Augenarzt für 8 Studierende zur Verfügung. Als Material wurden im Kurs, auf welchen sich die Evaluation bezieht, Schweinehaut und -augen verwendet. Mittlerweile werden Kunststoffaugen und -haut genutzt, und es können auch IVOM-Injektionen am Kunststoffauge durchgeführt werden.

## Ergebnisse

Es wurden 64 Evaluationsbögen von 8 Gruppen unterschiedlicher Dozenten zu je 8 Teilnehmern ausgewertet. Für die jeweiligen Aussagen bzw. Fragen konnten zwischen *n* = 59 und *n* = 61 Fragebögen einbezogen werden. Die Freitextkommentare wurden zur internen Evaluation des Veranstaltungserfolges herangezogen.

### Evaluation des Wetlabs

Das Wetlab zum mikrochirurgischen Nähen wurde nach Schulnoten mit einer 1,24 ± 0,5 (Mittelwert ± Standardabweichung) bewertet. Das Wetlab wurde als sehr hilfreich für das eigene Lernen bewertet, 1,27 ± 0,55 (1 = sehr hilfreich, 7 = gar nicht hilfreich). Der Aussage „Ich möchte gerne mehr Wetlab unter dem Mikroskop durchführen“ wurde deutlich zugestimmt mit 1,86 ± 1,28 (1 = stimme völlig zu, 7 = stimme gar nicht zu). Die Aussage „Ich möchte gerne mehr augenchirurgische Techniken erlernen“ wurde ebenfalls sehr positiv mit 2,02 ± 1,13 (1 = stimme völlig zu, 7 = stimme gar nicht zu) bewertet.

### Fragen zur Blockveranstaltung im Allgemeinen

**Figure 1:**
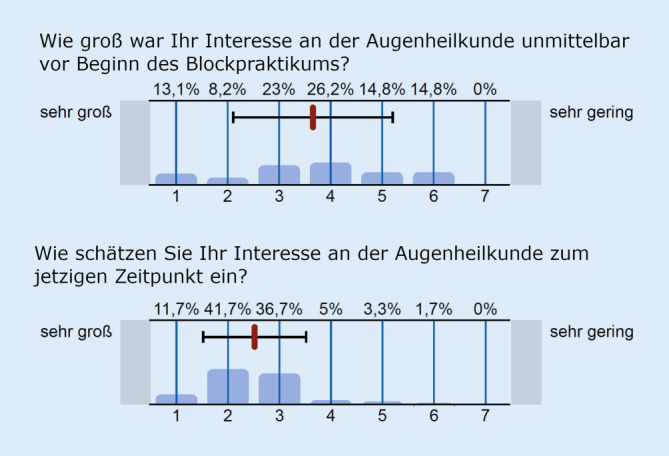

Das Blockpraktikum wurde nach Schulnoten mit einer 1,39 ± 0,62 bewertet. Zum Zeitpunkt der Evaluation nach Abschluss der Blockveranstaltung wurde das Interesse an der Augenheilkunde vor Beginn des Blockpraktikums mit 3,66 ± 1,55 (1 = sehr groß, 7 = sehr gering) angegeben, das Interesse an der Augenheilkunde nach dem Blockkurs mit 2,52 ± 1,00 (1 = sehr groß, 7 = sehr gering) größer bewertet. Dieser Unterschied war signifikant (*p* < 0,00001). Die Abb. 1 stellt die Verteilung der Interessenslage vor und nach der Veranstaltung dar.

## Diskussion

Die Ausbildung im Studium der Humanmedizin steht in Deutschland vor einem umfassenden Umbruch [1]. Reform- und Modellstudiengänge an zahlreichen Standorten entwickelten und evaluierten in den vergangenen Jahren moderne Lehrmethoden und stehen im Zeichen des „Kompetenzerwerbs“ im Gegensatz zum klassischeren Ansatz des vorrangigen Wissenserwerbs [6]. Im Zuge dieser Veränderungen sollte die Augenheilkunde nicht zurückbleiben, denn eine Auslastung der Weiterbildungskapazitäten ist im Sinne der zukünftigen Entwicklung des Faches, besonders hinsichtlich der hohen Facharztnachfrage bei gleichzeitig geringem Facharztangebot [4, 12]. Eine Diskrepanz in der Entscheidung zur Facharztweiterbildung in der Augenheilkunde tut sich derzeit im Verhältnis von interessierten und tatsächlich entschlossenen Studierenden auf: So beträgt der Anteil von Studierenden, welche sich tatsächlich für die Innere Medizin entscheiden würden, zu jenen, welche sich grundsätzlich vorstellen könnten, das Fach zu wählen 32,3 %. Für Kinder- und Jugendmedizin beträgt der Anteil beispielsweise 33,6 %, für Chirurgie 26,8 %. In der Augenheilkunde beträgt dieser Anteil lediglich 19,3 %. Zwar siedeln sich auch andere Fächer in diesem niedrigeren Bereich an, dennoch zeigt sich hieran, dass ungenutztes Potenzial besteht [2]. Das dargestellte Blockpraktikum soll solches Potenzial nutzen: Während ein makroskopischer Nahtkurs in den allgemeinchirurgischen Kursen wohl keine Seltenheit mehr darstellt, sind mikrochirurgische Angebote für Studierende noch rar, was sich auch in einer übersichtlichen Literaturlage zeigt. Es wurde jedoch bereits dargestellt, dass die Durchführung solcher Kurse für Studierende in Bezug auf die weitere berufliche Entwicklung Einfluss haben kann [5]. Auch andere Autoren empfehlen das Angebot solcher Kurse hinsichtlich anderer Aspekte, wie beispielsweise Evaluation von mikrochirurgischer Performance möglicher zukünftiger Kollegen oder wissenschaftlicher Mitarbeiter [9].

Einschränkend ist die schwierige Abbildbarkeit der konkreten individuellen Entscheidungsprozesse zum Weiterbildungsfach. Das gesteigerte Interesse kann im vorliegenden Studiendesign auch nicht klar aufgetrennt werden nach allgemeiner gelungener Exposition gegenüber dem Fach und der Steigerung durch die konkrete Durchführung praktischer mikrochirurgischer Tätigkeit im Wetlab. In Freitextantworten wurde dies jedoch explizit als besonders gelungen herausgehoben. Ob sich überwiegend besonders motivierte Studierende für den Kurs entschieden haben, können wir nicht mit Sicherheit sagen. Da das initiale Interesse an der Augenheilkunde jedoch als eher mittelmäßig angegeben wurde, gehen wir eher nicht davon aus. Gegebenenfalls haben sich chirurgisch interessierte Studierende vermehrt für das Blockpraktikum entschieden aufgrund des Wetlabs. Andere externe Faktoren, wie z. B. geplante Famulaturen oder Reisen während der vorlesungsfreien Zeit, spielen für diese Entscheidung jedoch gewiss auch eine Rolle.

Weiterhin wäre für zukünftige Evaluationen eine feinere Auftrennung nach Einzelaspekten des Kursaufbaus (theoretischer Teil, Patientenuntersuchung, Wetlab usw.) sinnvoll. Des Weiteren handelt es sich um eine noch zu erweiternde Fallzahl mit 64 Studierenden. Der Langzeiteffekt ist ebenfalls im Verlauf zu beurteilen. So ist nicht gesagt, dass die Begeisterung für das Wetlab dazu führt, dass sich vermehrt Studierende für Famulaturen, für ein praktisches Jahr oder später eine Weiterbildung im Fach Ophthalmologie entscheiden. Es stellt sicherlich aber eine gute Stütze in der Wegfindung zur Berufswahl für die Studierenden dar.

Wünschenswert wären weitere innovative Lehrprojekte in der Augenheilkunde, welche Vorzüge des Fachs darstellen und Kompetenzgewinn der Studierenden fördern. Vorstellbar sind nicht nur praktische Schulungen wie Nahtkurse oder behutsames Arbeiten mit organischem Gewebe, sondern auch die Stärkung interdisziplinärer Bezüge oder Forschungsseminare, welche wissenschaftlich interessierten Studierenden aktuelle Forschung zu ophthalmologischen oder systemischen Problemstellungen näherbringen.

Erwähnung finden sollte jedoch auch, dass selbst die besten Curricula das übergreifende gegenwärtige und zukünftige medizinische Versorgungsproblem nicht beheben können – letztendlich kämpfen auch die anderen Disziplinen um Nachwuchs. Deshalb sind neben Modernisierung des Studiums grundsätzlich Erweiterungen der Studienplatzkapazitäten und weitere Entlastungen wie mehr und qualifizierteres medizinisches Assistenzpersonal sowie bürokratischer Rückbau wünschenswert und notwendig [3, 7, 11].

## Ausblick

Die mikrochirurgische Ausbildung und das Praktikum sollten in Zukunft jedem Studierenden zugänglich werden.

## Fazit für die Praxis

- Die Einführung von mikrochirurgischen Wetlabs in die studentische Lehre ist sinnvoll und wird sehr positiv aufgenommen.
- Das Blockpraktikum, welches das mikrochirurgische Wetlab enthielt, steigerte das Interesse der Studierenden an der Augenheilkunde signifikant.
- Weitere innovative Lehrformen in den augenheilkundlichen Curricula sind sinnvoll und notwendig, um den Anschluss an eine moderne Lehre nicht zu verlieren.
